# Mitochondrial C11orf83 is a potent Antiviral Protein Independent of interferon production

**DOI:** 10.1038/srep44303

**Published:** 2017-04-18

**Authors:** Yun Yang, Shaoquan Xiong, Bei Cai, Hui Luo, E. Dong, Qiqi Li, Gaili Ji, Chengjian Zhao, Yanjun Wen, Yuquan Wei, Hanshuo Yang

**Affiliations:** 1 State Key Laboratory of Biotherapy and Cancer center/Collaborative Innovation Center for Biotherapy, West China Hospital, Sichuan University, 610041, Chengdu, China; 2 Department of Oncology, Affilicated Hospital of ChengDu University of Traditional Chinese Medicine, 610041, Chengdu, China; 3 Department of Laboratory Medicine/Research Center of Clinical Laboratory Medicine, West China Hospital, Sichuan University, 610041, Chengdu, China

## Abstract

Mitochondria have a central position in innate immune response via the adaptor protein MAVS in mitochondrial outer membrane to limit viral replication by inducing interferon production. Here, we reported that C11orf83, a component of complex III of electronic transfer chain in mitochondrial inner membrane, was a potent antiviral protein independent of interferon production. C11orf83 expression significantly increased in response to viral infection, and endows cells with stronger capability of inhibiting viral replication. Deletion of C11orf83 permits viral replication easier and cells were more vulnerable to viral killing. These effects mainly were mediated by triggering OAS3-RNase L system. C11orf83 overexpression induced higher transcription of OAS3, and knockdown either OAS3 or RNase L impaired the antiviral capability of C11orf83. Interestingly, the signaling from C11orf83 to OAS3-RNase L was independent of interferon production. Thus, our findings suggested a new antiviral mechanism by bridging cell metabolic machinery component with antiviral effectors.

The innate immune system limits viral replication during early stages of infections before the development of adaptive immunity^1^,^2^. The inducement of antiviral cytokines interferon (IFN) is an essential part of the antiviral response. Extensive research has provided detailed insights into the IFN signaling, however, much remains unknown about the mechanism that cell inhibits viral replication independent of IFN pathway.

Mitochondria functions as a signaling platform that is centrally positioned in the innate immune response against viral pathogens^3^. Retinoic acid-inducible gene I (RIG-I)-like receptors (RLRs) are one of four pattern-recognition receptors (PRRs) families of the innate immune system, which bind conserved molecular patterns that are shared by different kinds of microorganisms^4^. RLRs are expressed in the cytosol and are required for type I IFN and pro-inflammatory cytokine production in response to viral infection^5^,^6^,^7^. Mitochondrial antiviral signaling protein (MAVS), an adaptor molecule localized in the outer mitochondrial membrane (OMM), is essential for RLR signal transduction^8^. As downstream of RLRs, MAVS induces the production of type I IFN and pro-inflammatory cytokines during viral infection by activating NF-κB, IRF3 and IRF7 signaling cascades^8^,^9^. Mitochondrial cofactors take part in the regulation of RLR-MAVS signaling^10^,^11^,^12^. Moreover, mitochondrial dynamics govern antiviral signaling by enhancing mitochondria–MAMs interactions and RLR–MAVS signalosome formation around intracellular sites of viral infection^13^,^14^,^15^,^16^. Mitochondrial ROS is also shown to have a critical role in regulating RLR–MAVS signaling during viral infection^17^.

The 2′-5′-oligoadenylate synthetases (OASs) belongs to a nucleotidyltransferase superfamily^18^,^19^. The transcription of *OAS* genes is induced by both virus infection and IFN stimulation^20^,^21^. Upon binding to double-stranded RNA (dsRNA), OASs are activated through a conformational change to synthesize 2′-5′-phosphodiester-linked oligoadenylates (2-5As) and 2-5As acts as second messengers to activate RNase L^22^,^23^. Activation of RNase L leads to degradation of cellular and viral RNA and thereby suppresses viral replication^24^. Thus OASs-RNase L system offers protection against a wide range of RNA and DNA viruses. In humans, the OAS family consists of four members: OAS1, OAS2, OAS3, and OASL^25^. OAS1, OAS2, and OAS3 have 2′-5′-oligoadenylate synthetase activity, and OASL lacks this activity^26^,^27^. OAS3 is activated at a substantially lower concentration of dsRNA than OAS1, and synthesizes considerably longer 2-5As that are sufficient to activate RNase L intracellularly^19^,^28^.

Mammalian mitochondria may contain up to 1500 different proteins, and most of their functions have not been well revealed^29^. C11orf83, also named UQCC3, is a mitochondrial inner membrane protein. It is specifically associated with the complex III of the electron transport chain and is involved in the early stages of its assembly by stabilizing supercomplexes that contain complex III, especially the III_2_/IV supercomplex^30^. Patient cells with a homozygous c.59T > A missense mutation in C11orf83 have reduced complex III activity. The patient displays lactic acidosis, hypoglycemia, hypotonia, delayed development and severely delayed psychomotor development^31^. In the present study, we reported the identification of C11orf83 as a novel antiviral protein. Higher expression of C11orf83 responding to viral infection endows cells with stronger capability of inhibiting viral transcription, whereas the loss of C11orf83 expression renders viral replication easier in cells and cells were vulnerable to viral killing. Importantly, this effect mainly was mediated by triggering OAS3-RNase L system and was independent of interferons production, which is significantly different to MAVS.

## Results

### C11orf83 expression increases after virus infection

**Figure 1:**
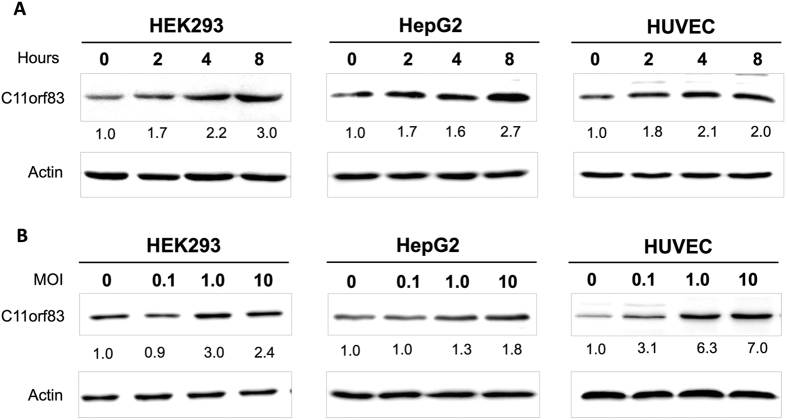
C11orf83 expression Increases after VSV infection. (**A**) Expression of C11orf83 in HEK293, HepG2 and HUVEC cells at different time points (0, 2, 4, 8 hours) after VSV infection (MOI = 1.0). (**B**) Expression of C11orf83 in HEK293, HepG2 and HUVEC cells at 8 hours post VSV infection with different MOI (0, 0.1, 1.0 and 10).

C11orf83 is a mitochondrial inner membrane protein^30^. To investigate whether C11orf83 is associated with virus infection, we used vesicular stomatitis virus (VSV) as the model virus^8^ and examined C11orf83 expression in human cells (HEK293, HUVEC and HepG2) at different time points after VSV infection. HEK293 is immortal human embryonic kidney cells, HUVEC is primary human endothelial cells, and HepG2 is malignant human hepatocellular carcinoma cells. The results clearly demonstrated that C11orf83 expression significantly increased after VSV infection. C11orf83 was upregulated early at 2 hours and higher at 4 and 8 hours after virus infection (Fig. 1A), suggesting C11orf83 is an early responsive protein during viral infection. Then, we detected C11orf83 expression responding to different amounts of VSV (MOI = 0, 0.1, 1.0 and 10), and found that more viral infection induced higher expression of C11orf83 (Fig. 1B). Moreover, qRT-PCR showed C11orf83 mRNA significantly increased at 2, 4, 8 hours post VSV infection (MOI = 1.0) in all three kinds of cells, and more amounts of VSV infection induced more significant increase of C11orf83 mRNA (Supplementary Fig. S1). These results suggested that the increased C11orf83 protein after VSV infection is most likely due to an increased transcription. Moreover, plaque assay and q-PCR results showed that the load of virus and viral RNA in cells was proportional to C11orf83 expression (Supplementary Fig. S2), indicating that C11orf83 is sensitive to virus load in cells.

### C11orf83 Is a Potent Antiviral Protein

**Figure 2:**
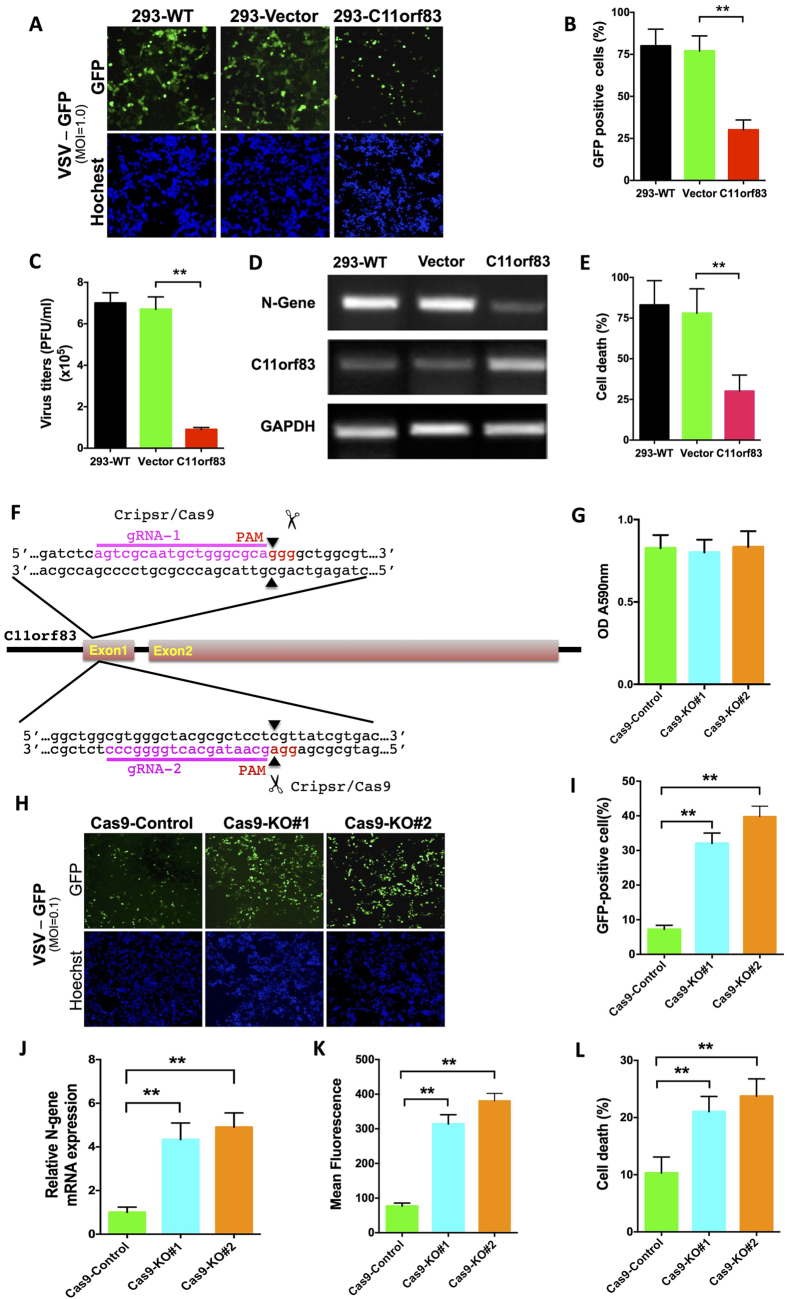
C11orf83 is a potent antiviral protein. (**A–E**) Effects of C11orf83 overexpression to rVSV-GFP replication in HEK293 cells. All analysis was performed at 12 hours post rVSV-GFP (MOI = 1.0) infection. **p < 0.01. **(A)** Representative imaging fluorescent shows the replication of rVSV-GFP in cells. (**B)** Quantitative analysis of GFP positive cells by using flow cytometry. **(C)** Quantitative analysis of virus titer in cells supernatants. **(D)** Transcription detection of C11orf83 and N gene of VSV using RT-PCR. **(E)** Quantitative analysis of VSV-induced cells death using Trypan Blue staining. (**F**) Graphical representation of the Human genomic loci of C11orf83 showed the targeting sites for Cas9, sgRNA, and mutation initiation site. The sgRNA targeting sequences are labeled in magenta. The PAM sequences are labeled in red. The mutation initiation sites are indicated by the black triangle and the DNA scissors. (**G**) Detection of cells viability using MTT.(**H–L)** Effects of the loss of C11orf83 to rVSV-GFP replication in HEK293 cells. All analysis was performed at 12 hours post rVSV-GFP (MOI = 0.1) infection. Cas9-Control, HEK293 cells transfected with Cas9 and control sgRNA. Cas9-KO#1 and Cas9-KO#2, HEK293 cells transfected with Cas9 and sgRNA#1 or sgRNA#2 as shown in F. **p < 0.01. (**H**) Representative imaging fluorescent shows the replication of rVSV-GFP in cells. **(I,K)** Quantitative analysis of GFP positive cells and mean fluorescence intensity in cells by using flow cytometry. **(J)** Quantitative RT-PCR results shows the relative change of N gene transcription. **(L)** Quantitative analysis of VSV-induced cells death using Trypan Blue staining.

Since C11orf83 is upregulated during virus infection, we examined whether C11orf83 participate in the anti-viral process in cells. VSV replicates rapidly in 293 cells^8^. We used a recombinant VSV line expressing GFP (rVSV-GFP) to demonstrate viral replication in infected cells^32^. C11orf83 Overexpression (Supplementary Fig. S3A) significant inhibited rVSV-GFP replication in 293 cells (Fig. 2A). Both fluorescent microscopic observations and flow cytometry analysis demonstrated the obviously decrease of GFP-positive cells in C11orf83-overexpressed 293 cells (Fig. 2A,B). Consistently, measurement of the viral titer in cells supernatants showed that C11orf83 overexpression made the decrease of viral titer by more than 7-fold as compared to control cells (Fig. 2C). N gene codes the nucleocapsid protein that is necessary to VSV. The detection to N gene at mRNA level clearly showed the much less of VSV mRNA than controls when C11orf83 was overexpressed (Fig. 2D). Infection of HEK293 cells by rVSV-GFP led to nearly complete killing of the cells within 24 hours (MOI = 0.01). However, only about 30% cells were killed by rVSV-GFP infection in C11orf83-overexpression cells (Fig. 2E).

Next, we deleted endogenous C11orf83 expression by using CRISPR-Cas9 system (Supplementary Fig. S3B,C). We used two sgRNAs that effectively cleaved C11orf83 gene and produced mutations in C11orf83 exon 1 (Fig. 2F). The C11orf83 deletion did not caused any obvious effects to cell growth (Fig. 2G), but fluorescence and N gene detection and plaque assay to rVSV-GFP demonstrated that C11orf83 deletion leaded to an increase of viral replication by about 5-fold as compared to control cells (Fig. 2H–K and Supplementary Fig. S4). Consequently, C11orf83 deficiency greatly sensitized cell killing by rVSV-GFP at a much lower MOI (0.01) (Fig. 2L). Similarly, viral replication in C11orf83-deleted human HepG2 cell also markedly increased (Supplementary Fig. S5). The plaque assay to VSV growth kinetic in control and C11orf83-KO cells demonstrated that C11orf83 significantly inhibited VSV replication within 24 hours post viral infection (Supplementary Fig. S6). Importantly, the effects caused by Crispr/Cas9 could be partial rescued by C11orf83 reconstitution (Supplementary Fig. S7). Together, these results show that C11orf83 is a pivotal cellular antiviral protein.

### C11orf83 inhibits virus replication through OAS3-RNase L system

**Figure 3:**
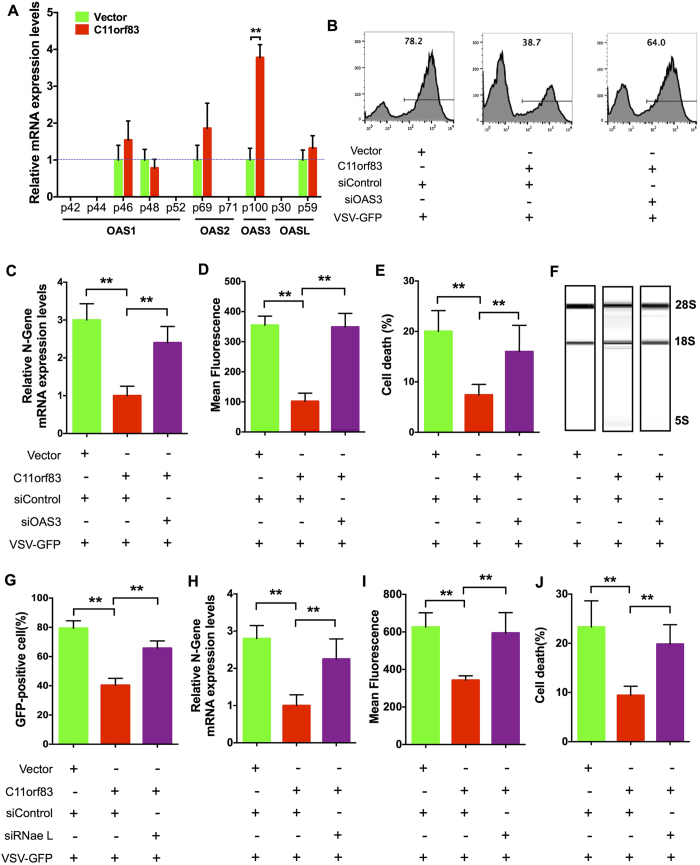
C11orf83 inhibits VSV replication through OAS3-RNaseL system. (**A**) qPCR analysis of OAS variants in C11orf83 overexpressed HEK293 cells. **p < 0.01. (**B–F**) Effects of the loss of OAS3 to rVSV-GFP replication in C11orf83 overexpressed HEK293 cells. HEK293 cells were transfected with pcDNA3.1 (vector) or pcDNA3.1-C11orf83, and 48 hours later cells were transfected with siControl or siOAS3. In the next day, cells were infected with rVSV-GFP (MOI = 1.0) and 12 hours later assays were performed. **p < 0.01. (**B,D**) Quantitative analysis of GFP positive cells and mean fluorescence intensity in cells by using flow cytometry. (**C**) Quantitative RT-PCR results shows the relative change of N gene transcription. (**E**) Quantitative analysis of VSV-induced cells death using Trypan Blue staining. (**F**) RNA integrity analysis using Agilent Bioanalyzer 2100. (**G–J**) Effects of the loss of RNase L to rVSV-GFP replication in C11orf83 overexpressed HEK293 cells. HEK293 cells were transfected with pcDNA3.1 (vector) or pcDNA3.1-C11orf83, and 48 hours later cells were transfected with siControl or siRNase L. In the next day, cells were infected with rVSV-GFP (MOI = 1.0) and 12 hours later assays were performed. **p < 0.01. **(G,I)** Quantitative analysis of GFP positive cells and mean fluorescence intensity in cells by using flow cytometry. **(H)** Quantitative RT-PCR results shows the relative change of N gene transcription. **(J)** Quantitative analysis of VSV-induced cells death using Trypan Blue staining.

OASs-RNase L system is one of the most important cellular antiviral mechanism, providing protection against a wide range of RNA and DNA viruses. The 42-kDa OAS1 consists of one OAS domain, while the 69-kDa OAS2 consists of two OAS domains and the 120-kDa OAS3 consists of three OAS domains^25^,^33^. To investigate whether the antiviral effect of C11orf83 is through OASs-RNase L system, we examined the expression of OAS family four members (OAS1, OAS2, OAS3, and OASL) at the mRNA level in HEK293 cells that only overexpressed C11orf83 but no viral infection. qPCR results revealed that C11orf83 resulted in the upregulation of OAS1, OAS3 and OASL expression, whereas OAS3 (p100) had the most significantly alternation by about 4 folds of increase (Fig. 3A). Also, OAS3 transcription dramatically increased after VSV infection, and is significantly impaired when c11orf83 is absent (Supplementary Fig. S8). These suggested that OAS3 might play an important role during antiviral process triggered by C11orf83.

OAS family is the activator of endoribonuclease RNase L and RNase L is known to terminate virus replication by cleaving viral and cellular RNAs. We found that when endogenous OAS3 or RNase L was silenced in C11orf83-overexperssed HEK293 cells by using small interfering RNA (siRNA) (Supplementary Fig. S9)^34^, C11orf83 lost the anti-viral capability evidenced by the analysis to viral green fluorescence, viral N gene, viral titer (plaque analysis), cell death and mRNA degradation detection (Fig. 3B–J and Supplementary Fig. S10). All siRNAs had no obvious toxic affects to cells (Supplementary Fig. S11). OAS3 knockdown did not further affect viral titer in c11orf83KO cells, suggesting OAS3 is the downstream of C11orf83 (Supplementary Fig. S12). These results together indicated that c11orf83 inhibits virus replication through the OAS3-RNase L system, and were confirmed in HepG2 cells (Supplementary Fig. S13A–I).

### The antiviral effect of C11orf83 is independent of IFN production

**Figure 4:**
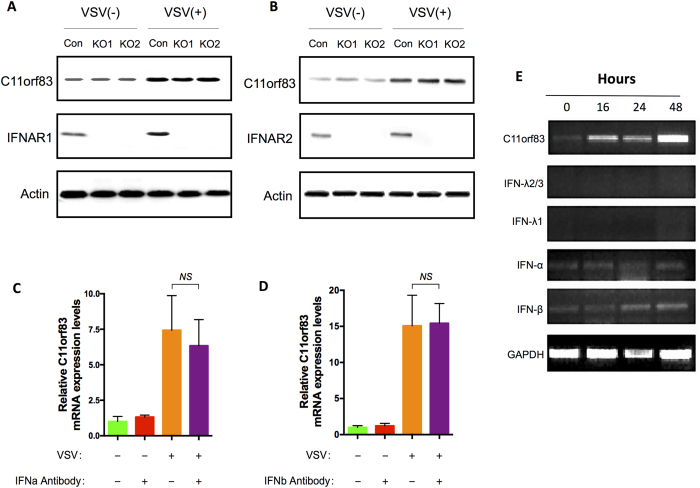
C11orf83 is not related to IFNs. (**A,B**) VSV infection upregulating c11orf83 expression in IFNR1- and IFNR2-deficient cells. (**C,D**) VSV infection upregulating c11orf83 expression in the presence of type I IFN (IFNα and IFNβ) neutralizing antibodies. (**E**) RT-PCR analysis to the transcription of C11orf83 and IFNs (IFN-λ2/3, IFN-λ1, IFN-α and IFN-β) in HEK293 cells at 0, 16, 24 and 48 hours after the transfection of C11orf83 gene.

Interferons (IFNs) play important roles during anti-viral process by inducing expression of interferon stimulated genes (ISGs). To reveal whether C11orf83 is an ISG, we detected the kinetics of C11orf83 expression upon type I, type II and type III IFNs at early and late time points post stimulation. The results clearly showed that c11orf83 expressions were not significantly affected by type I, type II and type III IFNs (Supplementary Fig. S14). Then, we detected c11orf83 expression in IFNR1 or IFNR2 knock-out cells with or without viral infection, and examined transcriptional alternation of c11orf83 in the presence of type I IFN (IFNα and IFNβ) neutralizing antibodies (Supplementary Fig. S15 and Fig. 4). We found that neither IFN receptors deficiency nor IFN blocking impaired viral infection-induced c11orf83 expression (Fig. 4A–D). The results indicated that C11orf83 is induced in an interferon independent manner, suggesting c11orf83 is not an ISG itself. Moreover, C11orf83 overexpression also did not trigger expression of IFNα, IFNβ and IFNγ (Fig. 4E).

**Figure 5:**
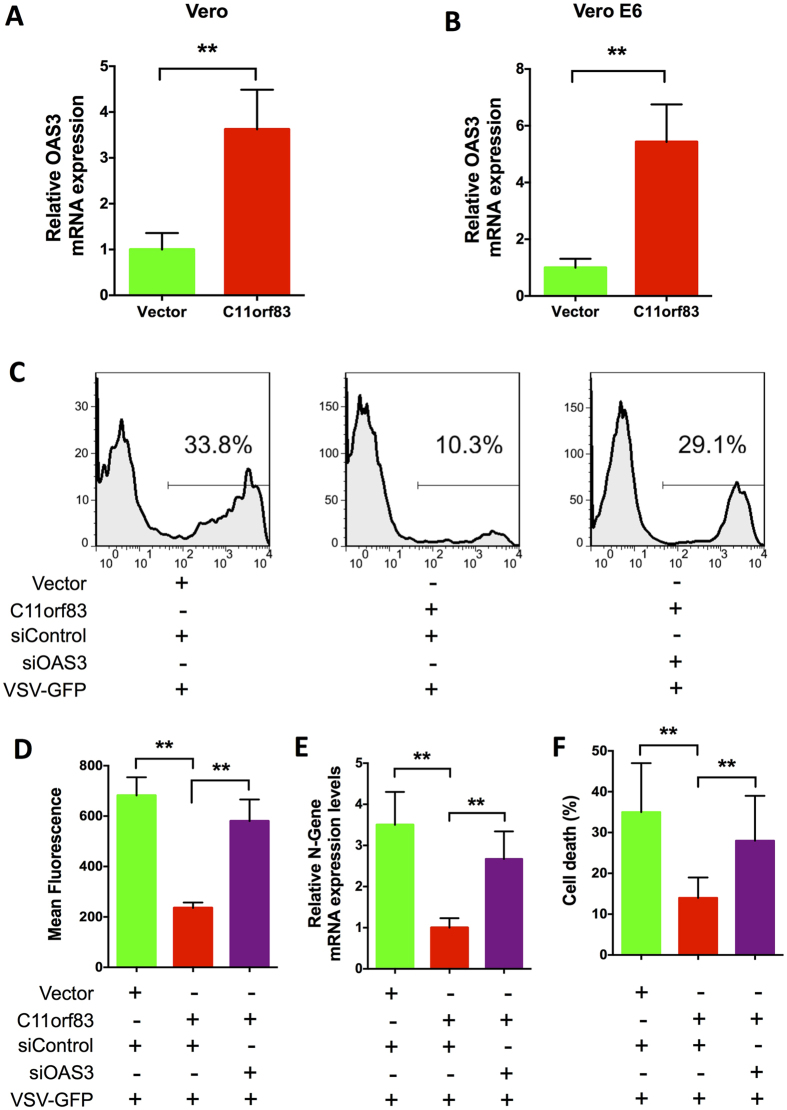
C11orf83 inhibits VSV replication through OAS3-RNaseL system in the absence of IFNs production. (**A,B**) qPCR analysis to the expression of OAS3 in the mRNA level in IFN-deficient Vero (B) and Vero E6 (C) cells. Vero and Vero E6 cells were transfected with pcDNA3.1 (vector) or pcDNA3.1-C11orf83-monkey. **p < 0.01. (**C–F**) Effects of the loss of OAS3 to rVSV-GFP replication in C11orf83 overexpressed Vero cells. Vero cells were transfected with pcDNA3.1 (vector) or pcDNA3.1-C11orf83-monkey, and 48 hours later cells were transfected with siControl or siOAS3. In the next day, cells were infected with rVSV-GFP (MOI = 0.1) and 12 hours later assays were performed. **p < 0.01. **(C,D)** Quantitative analysis of GFP positive cells and mean fluorescence intensity in cells by using flow cytometry. **(E)** Quantitative RT-PCR results shows the relative change of N gene transcription. **(F)** Quantitative analysis of VSV-induced cells death using Trypan Blue staining.

OASs are a family of interferon-stimulated genes (ISGs). The transcription of OAS family genes can be induced by TypeI IFN stimulation. To further reveal whether C11orf83 upregulating OAS3 depends on TypeI IFN or not, we examined OAS3 expression in Vero and Vero E6 cells that are intrinsically deficient in type I IFNs production because lack the gene cluster for type I IFN^35^. We found that overexpression of C1H11orf83 (the *Chlorocebus sabaeus* homology of human c11orf83) still induced the upregulation of OAS3 (Fig. 5A,B) and markedly decreased viral replication in Vero and Vero E6 cells (Fig. 5C). When endogenous OAS3 was knocked-down using siRNA, C11orf83 lost the capability of inhibiting rVSV-GFP replication in Vero cells (MOI = 0.1) (Fig. 5D–F).

**Figure 6:**
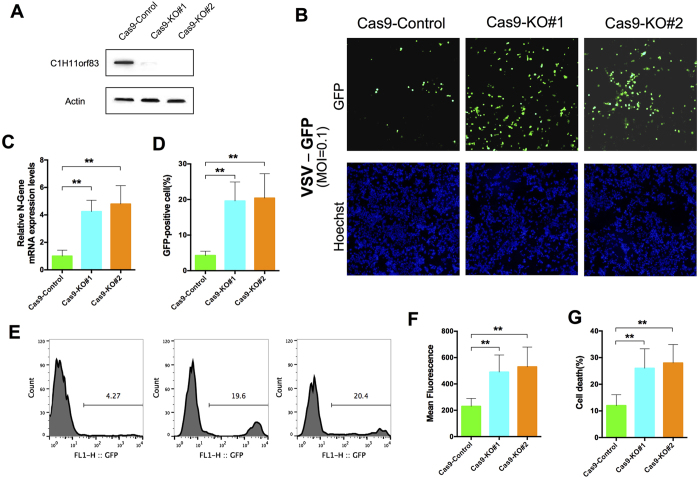
C11orf83 plays an important role in inhibiting VSV replication in the absence of IFNs production. (**A**) The absence of C11orf83 protein in Vero cells by Crispr-Cas9 system. (**B–G**) Effects of c11orf83 deletion to rVSV-GFP replication in Vero cells. (**B**) Representative imaging fluorescent shows the replication of rVSV-GFP in cells. (**C**) Quantitative RT-PCR results showing the relative change of N gene transcription. (**D–F**) Quantitative analysis of GFP positive cells and mean fluorescence intensity in cells by using flow cytometry. (**G**) Quantitative analysis of VSV-induced cells death using Trypan Blue staining.

In addition, we deleted endogenous C11orf83 expression in Vero cells by using CRISPR-Cas9 system (Fig. 6). We used two sgRNAs that effectively cleaved C11orf83 gene in exon 1 and terminated C11orf83 protein expression in Vero cells (Supplementary Fig. S16 and Fig. 6A). Viral fluorescence, N gene detection, plaque assay and cell death analysis demonstrated that C11orf83 deletion leaded to the significant increase of viral replication in Vero cells (Fig. 6B–G). Together, these results indicated that C11orf83 functions as an antiviral protein independent of IFNs signaling.

## Discussion

Mitochondria are dynamic double-membrane-bound organelles in eukaryotic cells. Mitochondria are involved in a broad range of cellular processes, such as ATP generation, apoptosis, calcium homeostasis, and the biosynthesis of amino acids, lipids and nucleotides. Recent research has unveiled that mitochondria participate in innate immune responses, such as antiviral signaling, anti-bacterial immunity and sterile inflammation. MAVS, a mitochondrial antiviral signaling protein (also named IFNβ promoter stimulator 1 (IPS1), CARD adaptor inducing IFNβ (CARDIF) or virus-induced signalling adaptor (VISA)), localized in the outer mitochondrial membrane and signaling downstream of RLRs during viral infection^8^,^9^,^36^,^37^. Viral dsRNA recruits the RIG-I and MDA-5 to interact with MAVS directly or indirectly. The signal of viral replication thus is transmitted to the mitochondria, and further triggers antiviral responses through activation of NF-κB, IRF3 and IRF7 signaling^8^,^9^,^36^,^37^. In addition, many common innate immune signaling molecules have been shown to be downstream of MAVS^6^. Therefore, mitochondria are centrally positioned in the innate immune response functioning as signaling platforms against viral pathogens.

Mitochondrial own genome encodes 13 proteins of the oxidative phosphorylation machinery. However, mitochondrial proteome comprises about 1500 proteins, most of which are encoded by nuclear genome. C11orf83 is a nuclear genome coded small protein, targeted and anchored in the mitochondrial inner membrane by its N-terminal section. C11orf83 is currently known as an assembly factor of Complex III of the electron transport chain and is required for proper mitochondrial morphology and function. C11orf83-deficient cells displayed complex III assembly deficiency and abnormal mitochondria with an impaired oxidative phosphorylation^30^,^31^. In the present study, we found that C11orf83 also is sensitive to viral infection, which was rapidly upregulated when cells were infected by VSV. Higher expression of C11orf83 endows cells with stronger immunity to viral infection, whereas the loss of C11orf83 expression renders VSV replication easier in cells and cells were vulnerable to viral killing. Thus, we propose that C11orf83 is novel antiviral protein in mitochondria. At least to our known, our findings also firstly bridge mitochondrial energy metabolism (oxidative phosphorylation) machinery to antiviral mechanism in innate immune response.

Detection of virus-derived nucleic acids plays a central role in initiating antiviral immunity. Nucleic acid recognition by PRRs results in the secretion of type I interferon (IFN) cytokines and IFN-stimulated genes (ISGs), which function to impede viral replication. We have known two kinds of cellular sensors to viral infection: first is cGMP-AMP synthase (cGAS) as a DNA sensor that recognizes cytosolic DNA and subsequently produces cGMP-AMP (cGAMP) to trigger interferon production^38^,^39^. Second are RNA sensors including cytoplasmic RNA helicase RIG-I as the sensor of 5′-triphosphorylated RNA and MDA5 as the sensor of long double-stranded RNA^40^. C11orf83 localizes in mitochondrial inner membrane as an important component of complex III^30^, it does not seem to be reasonable that C11orf83 functions as an adapter molecule liking MAVS. So, we would like to believe that C11orf83 might represent a novel kind of cellular sensor, a metabolic sensor, to virus infection.

Type I interferon responses are considered the primary means by which viral infections are controlled in mammal cells. In response to virus infection, RIG-I senses viral RNA and activates mitochondrial MAVS, the adaptor protein that stimulates downstream signaling pathways leading to type I interferon production. Then interferon induces the expression of antiviral effectors, Interferon-stimulated gene (ISG) such as OASs and Mx, to restrict virus propagation in cells. Mitochondrial dynamics and mitochondrial ROS also participate in governing antiviral process by modulating RLR–MAVS signaling. C11orf83 is a potent antiviral protein in mitochondrial inner membrane. Different to above known mechanisms, C11orf83 induces higher expression of interferon effectors OAS3 and results in the cleavage of cellular and viral RNA. Although we do not know how C11orf83 induces the upregulation of OAS3, we show that this process is independent of interferons production. To the best of our knowledge, this the first report that a mitochondrial protein triggers the activation of antiviral effector system of OAS3-RNase L. Together, our studies have uncovered a new mechanism of mitochondria in innate immunity against viral infection, but much more work is required in the future to elucidate the biochemical mechanism of C11orf83 signaling, at least including the relationship of C11orf83 to MAVS, the signaling that mediates C11orf83 activating OAS3-RNase L system, and whether C11orf83 can efficiently prevent the production of viral proteins by inhibiting translational machinery.

## Materials and Methods

### Cells and Virus

HEK293, HepG2, HUVEC-CS, Vero and VeroE6 cells were grown in DMEM (Gibco) supplemented with 10% of heat-inactivated FBS and 1% penicillin and streptomycin at 5% CO_2_ and 37 °C.

The Vesicular Stomatitis Virus (Indiana strain) and the VSV-GFP recombinant (rVSV-GFP) virus were kindly provided by Dr. Chunsheng Dong in Soochow University. *GFP* was fused to the coding sequence of the G protein in VSV genome. VSV and rVSV–GFP replication in infected cells was indicated by green fluorescence.

### Plasmid Constructs

The coding sequence (CDS) of human and monkey (*Chlorocebus sabaeus*) C11orf83 and its mutant of human C11orf83 cDNA was inserted into the pcDNA3.1 expression vector between the restriction sites BamH I and XhoI. The recombinant constructs were named pcDNA3.1-C11orf83, pcDNA3.1-C11orf83-Mutant, and pcDNA3.1- C11orf83-monkey. The empty pcDNA3.1 was used as a control.

To construct a recombinant eukaryotic vector bearing the fusion gene of human C11orf83 gene and EGFP (enhanced green fluorescent protein), and to transfect it into HEK293T cells for its subcellular localization, the pEGFP-N1 was used to construct pEGFP-N1-C11orf83 and pEGFP-N1-C11orf83-Mutant between the restriction sites HindIII.

CRISPR/Cas9 system was used to disrupt the endogenous C11orf83, IFNAR1 and IFNAR2 in human HEK293T or HepG2 cells, and monkey (*Chlorocebus sabaeus*) Vero cells. The annealed double-stranded single-guide RNAs (sgRNAs) downstream of the C11orf83 start codon were cloned into LentiCRISPRv2 (Addgene) by BsmB I (NEB). Two sgRNAs targeting human C11orf83 (#1 agtcgcaatgctgggcgca and #2 cccggggtcacgataacg), two sgRNAs targeting human IFNAR1(#1 gacaggagcgatgagtctgt and #2 gcaggagcgatgagtctgtc), two sgRNAs targeting human IFNAR2(#1 gttccggtccatcttatcat and #2 gtttccggtccatcttatca) and two sgRNAs targetting monkey (*Chlorocebus sabaeus*) C11orf83 (#1 gctgatcacagtcgcagtgc and #2 gcagtcgcagtgctgggcgc) were effective to produce mutation of respective gene in genomic DNA.

### Transfection

For transient transfection, cells (HEK293, HepG2, Vero or Vero E6) were seeded in 6-well plates and were grown to 80–90% confluence. An aliquot of plasmid DNA (5 μg) was mixed with Lipofectamine 3000 reagent (7.5 μL) (Life Technology) and added to cells as suggested by the manufacturer. Twelve hours after incubation, the cells were placed in serum-containing medium and further incubated at 37 °C.

### Viral infection and plaque assays

VSV and rVSV-GFP were propagated and amplified by infecting the monolayer of HEK293 cells. The supernatant was harvested 24 hours later and clarified by centrifugation.

Viral titers were determined by evaluating plaque assays in HEK293 cells. The HEK293 cells (1 × 10^5^) were transfected with the indicated plasmids for 48 h and seeded into 24-well plate. The viral sample were diluted 1:10^1~6^ and infect confluent HEK293 cells seeded before. At 1 h after infection, uninfected viruses were washed three times. Then, 3% methylcellulose was overlaid. At 24 hours after infection, overlay was removed and cells were fixed with 4% formaldehyde for 20 min and stained with 0.25% crystal violet. Plaques were counted and multiplied by the dilution factor to determine viral titer as LOG_10_ (pfu/ml).

### Western blotting

Cells were harvested and lysed with RIPA (20 mM Tris PH7.5, 150 mM NaCl, 1% Triton X-100, 2.5 mM sodium pyrophosphate, 1 mM EDTA, 1% Na3VO4, 0.5 μg/ml leupeptin, 1 mM PMSF). For immunoblotting, proteins from whole-cell lysates were resolved by 10 or 12% sodium dodecyl sulfate-polyacrylamide gel electrophoresis (SDS-PAGE) and then transferred to PVDF membranes. C11orf83 antibodies (Abmart, China) were used at 1:1000 and secondary antibodies conjugated with horseradish peroxidase (HRP) were used at 1:1000 dilutions in 5% non-fat dry milk. After the final washing, PVDF membranes were exposed for an enhanced chemiluminescence assay using the Gel imaging instrument (BIO-RAD ChemiDoc MP). The gray value of all the bands is measured by IMAGEJ.

### qPCR

Total RNA was isolated with the TRIZOL reagent (Life technology) and reverse transcribed with PrimeScript RT reagent Kit with gDNA Eraser Kit (Takara RR047a). Quantitative PCR analysis was performed using a BIO-RAD CFX96 Real-Time PCR System (BIO-RAD) with SYBR Green qPCR Master Mix for all the target genes (Takara, 638320). Primers for ACTB or GAPDH were used as the internal control. All of the primer sequences are listed in Table 1.

### siRNA

Cells were trypsinized and incubated overnight to achieve 60–70% confluence before siRNA transfection. The siRNA sequences were respectively shown below (Bréhin, A. C. *et al*.^34^): OAS3 siRNA (Ribobio Corporation; 200 nM; sense, 5′-UUUGGU GCCAGAACUGAGC(dTdT)-3′; antisense, 5′-GCUCAGUUCUGGCACCAAATT (dTdT)-3′), RNase L siRNA (Ribobio Corporation; 200 nM; sense, 5′-AGUGGA CGACUAAGAUUAATT(dTdT)-3′; antisense, 5′-UUAAUCUUAGUCGUCCACU TG(dTdT)-3′) and negative control siRNA (Ribobio Corporation) were mixed with Lipofectamine 3000 (Invitrogen). The cells were incubated with the transfection mixture for 48 h and then rinsed with DMEM containing 10% fetal bovine serum. The cells were then infected with rVSV-GFP.

### Flow Cytometry

Single-colour FCM was used to determine the percentage of GFP positive cells. Briefly, cells were collected and suspended in PBS and analyzed by flow cytometer (BD FACSCalibur) with excitation and emission wavelengths at 488 nm and 530 nm, respectively. The data were analyzed using FlowJo 10.0.7 software.

### Confocal microscopy

For experiments confirming proper localization of the C11orf83 and mitochondria, HEK293T expressing pEGFP-N1-C11orf83 or pEGFP-N1-C11orf83-Mutant were seeded into six-well plates (Corning). After VSV infection, cells were incubated with 25 nM MitoTracker Red FM (Thermo Fisher Scientific M22425) for 30 minutes and were fixed in 2% paraformaldehyde for 20 min. Cells were then washed three times with PBS-T (PBS containing 0.05% Tween-20) and were then stained with DAPI at 25 °C for 10 min. Images were acquired under a LEICA TCS SP5II confocal microscope.

### RNA fragment analysis

HEK293 Cells first were transfected by pcDNA3.1, pcDNA3.1-C11orf83, OAS3-siRNA or control siRNA, respectively. 24 hours later cells were infected with VSV (MOI = 1.0) for twelve hours, and then cell total RNA was isolated with Trizol (Life Technologies) and rRNA degradation was monitored in RNA chips with an Agilent 2100 bioanalyzer as the standard protocol.

### Statistical analysis

Student’s t-test was used in all cellular experiments and the results from three independent experiments are presented as mean SEM. In addition, all the mRNA expression data represent the mean values with standard error of the mean (SEM). The differences in mRNA expression data were compared using the ratio t-test. All statistical analyses were performed using the GraphPad Prism software. Asterisks denote statistical significance as follows: ns, P > 0.05; *P ≤ 0.05; **P ≤ 0.01.

## Additional Information

**How to cite this article:** Yang, Y. *et al*. Mitochondrial C11orf83 is a potent Antiviral Protein Independent of interferon production. *Sci. Rep.* **7**, 44303; doi: 10.1038/srep44303 (2017).

**Publisher's note:** Springer Nature remains neutral with regard to jurisdictional claims in published maps and institutional affiliations.

## Supplementary Material

Supplementary Figures

## Figures and Tables

**Table 1:** Primers for qPCR.

Genes	Primer sequence	
	Forward	Reverse
C11orf83	ATGGATTCCTTGCGGAAAAT	ACCATCCAGTTCTTCCTCCA
N-Gene	CTAAACTACCGGCCAATGAGGAT	TCCGCTAACCTTGCTGAGAT
OAS1-p42	ATGATGGATCTCAGAAATACCC	TCAAGCTTCATGGAGAGGG
OAS1-p44	ATGATGGATCTCAGAAATACCC	CTAAGCAACCTGGAAACTATAGGATAA
OAS1-p46	ATGATGGATCTCAGAAATACCC	TCAGAGGATGGTGCAGGTC
OAS1-p48	ATGATGGATCTCAGAAATACCC	TCAGGAGACCTGGGTTCTGTC
OAS1-p52	ATGATGGATCTCAGAAATACCC	TTATCTATGATAGGATAGAGGGCATAGA
OAS2-p69	ATGGGAAATGGGGAGTCCC	TTAGATGACTTTTACCGGCACTTT
OAS2-p71	ATGGGAAATGGGGAGTCCC	CTAGAAGTTTCTTTTAGAATTATTATTCAGGA
OAS3 p100	CTTGAGGACTGGATGGATGTTAG	ACTGTTGAGGAGGGTAGAGTAG
OASL p59	ATGGCACTGATGCAGGAACTG	CTAACTGGCTGGAAACAGAGCC
OASL p30	ATGGCACTGATGCAGGAACTG	TCATAGCAACAGTCCTGTTTCAGG
RNase L	CCTGGGCCTTCTGAACATT	TGTTCTGGTAGAAATTGCCTCT
Actin	GGACCTGACTGACTACCTCAT	CGTAGCACAGCTTCTCCTTAAT
IFN-α	TCCATGAGATGATCCAGCAG	ATTTCTGCTCTGACAACCTCC
IFN-β	GACGCCGCATTGACCATCTA	CCTTAGGATTTCCACTCTGACT
IFN-λ1	CTGGAGGCATCTGTCACCTT	TGGGTTGACGTTCTCAGACA
IFN-λ2/3	GCCTCTGTCACCTTCAACCTC	GGAGGGTCAGACACACAGGT
OAS3(Vero)	CTGGTCTGAGCCTCAAGTTTC	GGCTAACATCCATCCAGTCTTC
GAPDH(Vero)	GGTGTGAACCATGAGAAGTATGA	GAGTCCTTCCACGATACCAAAG
GAPDH	ACCACAGTCCATGCCATCAC	TCCACCACCCTGTTGCTGTA
