# Supplementary material for: Mitochondrial C11orf83 is a potent Antiviral Protein Independent of interferon production
Source: Sci Rep. 2017 Apr 18;7:44303. doi: 10.1038/srep44303 (PMC5394693; doi:10.1038/srep44303)
Supplement: Supplementary Figures [file srep44303-s1.pdf]

Supplementary Figures

Mitochondrial C11orf83 is a potent Antiviral Protein Independent of interferon production

Yun Yang[1,†], Shaoquan Xiong[1,2,†], Bei Cai[3,†], Hui Luo[1], E Dong[1], Qiqi Li[1], Gaili Ji[1], Chengjian Zhao[1], Yanjun Wen[1], Yuquan Wei[1], Hanshuo Yang[1,*]

1. State Key Laboratory of Biotherapy and Cancer center/Collaborative Innovation Center for Biotherapy, West China Hospital, Sichuan University, 610041, Chengdu, China.
2. Department of Oncology, Affilicated Hospital of ChengDu University of Traditional Chinese Medicine, 610041, Chengdu, China.
3. Department of Laboratory Medicine/Research Center of Clinical Laboratory Medicine, West China Hospital, Sichuan University610041, Chengdu, China.

† These authors contributed equally to this work.

* Correspondence: yhansh@scu.edu.cn

Supplementary Figure1

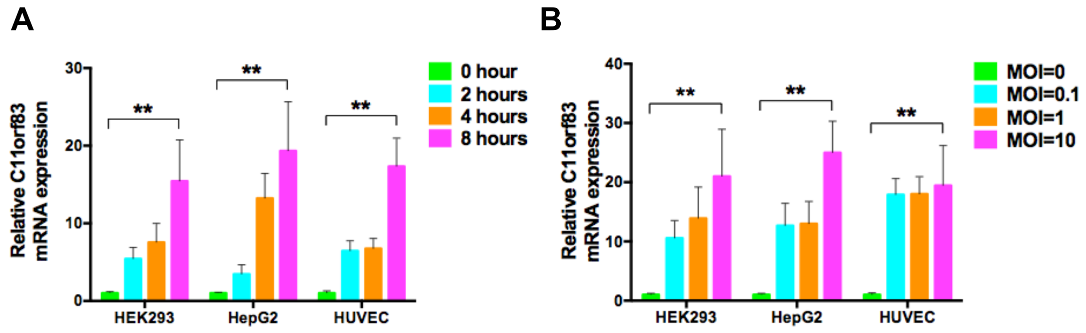

Fig.S1. qRT-PCR detecting C11orf83 transcription at different time points (0,2,4,8 hours) after VSV infection (MOI=1.0) **(A)** or at 8 hours post VSV infection with different MOI (0, 0.1 ,1.0 and 10) **(B)** in HEK293, HepG2 and HUVEC cells. **, p<0.01. NS, No Significance.

Supplementary Figure 2

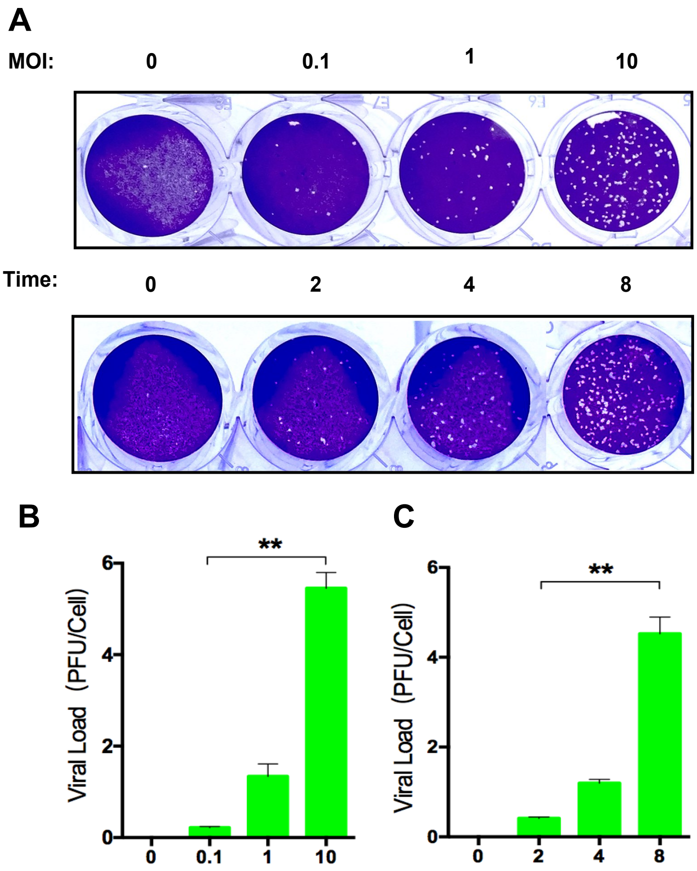

Fig.S2. (A) Plaque assay of viral titers at 8 hours post VSV infection with different MOI (0, 0.1 ,1.0 and 10) in HEK293 and plaque assay of viral titers at different time points (0,2,4,8 hours) after VSV infection (MOI=1.0) in HEK293. **(B)** Quantification of viral load (PFU/cell) at 8 hours post VSV infection with different MOI(0, 0.1 ,1.0 and 10) in HEK293. **(C)** Quantification of viral load (PFU/cell) at different time points (0,2,4,8 hours) after VSV infection (MOI=1.0) in HEK293. **, p<0.01.

Supplementary Figure 3

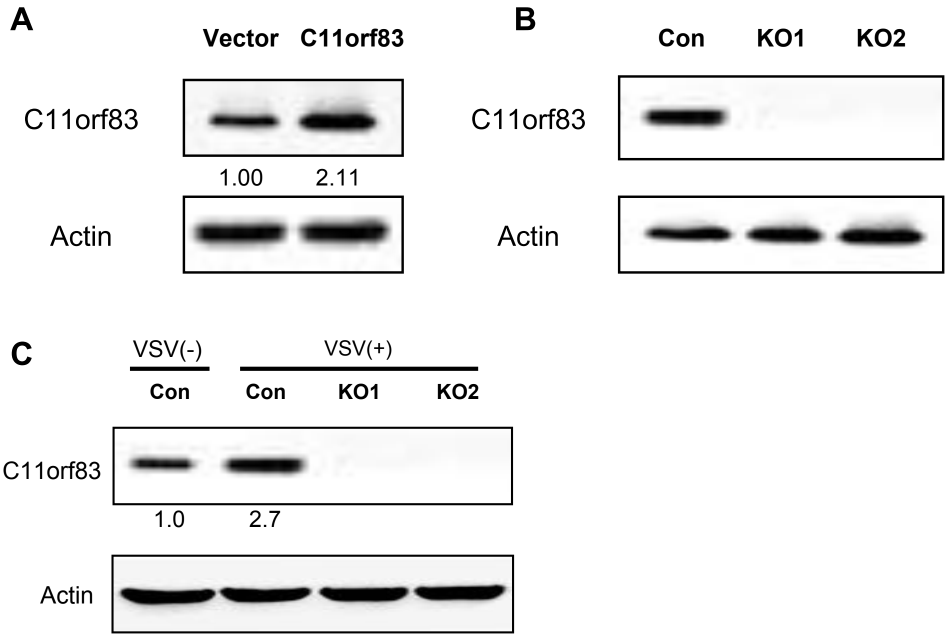

Fig.S3. (A) Expression of C11orf83 protein in HEK293 cells transfected with pcDNA3.1 and pcDNA3.1-C11orf83. **(B)** Expression of C11orf83 protein in HEK293 and C11orf83-deficient HEK293 cells. C11orf83-deficient HEK293 cells were established using CRISPR/Cas9 system. Con: Cas9-Control; KO1: Cas9-KO#1; KO2: Cas9-KO#2. **(C)** Expression of C11orf83 protein in C11orf83-deficient cells at basal levels and after VSV infection (MOI=10).

Supplementary Figure 4

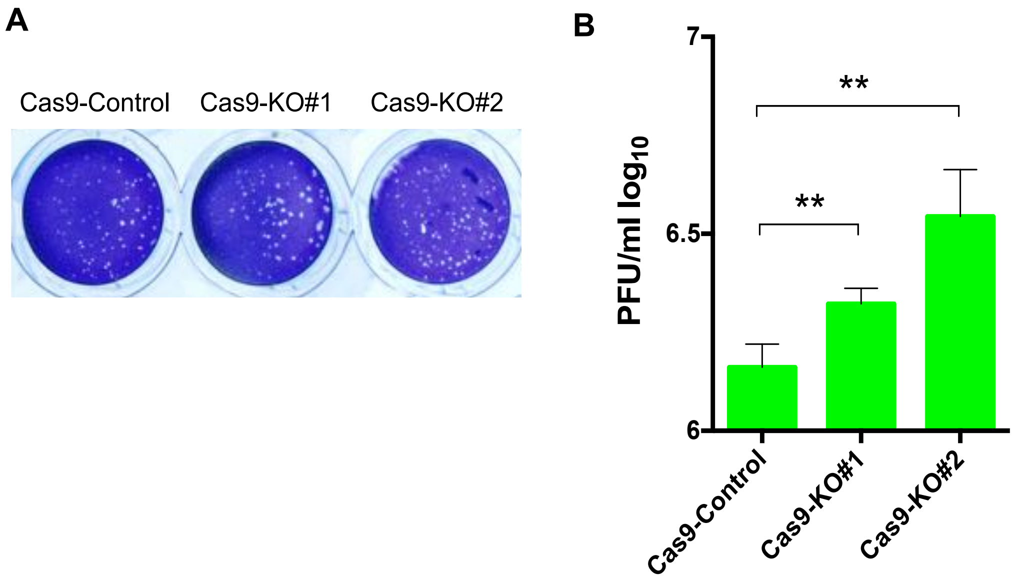

Fig.S4. (A) Plaque assay of viral titers in C11orf83-deficient HEK293 cells at 12 hours after VSV infection (MOI=0.1). **(B)** Quantification of plaque assay to viral titers in C11orf83-deficient HEK293 cells **, p<0.01.

Supplementary Figure 5

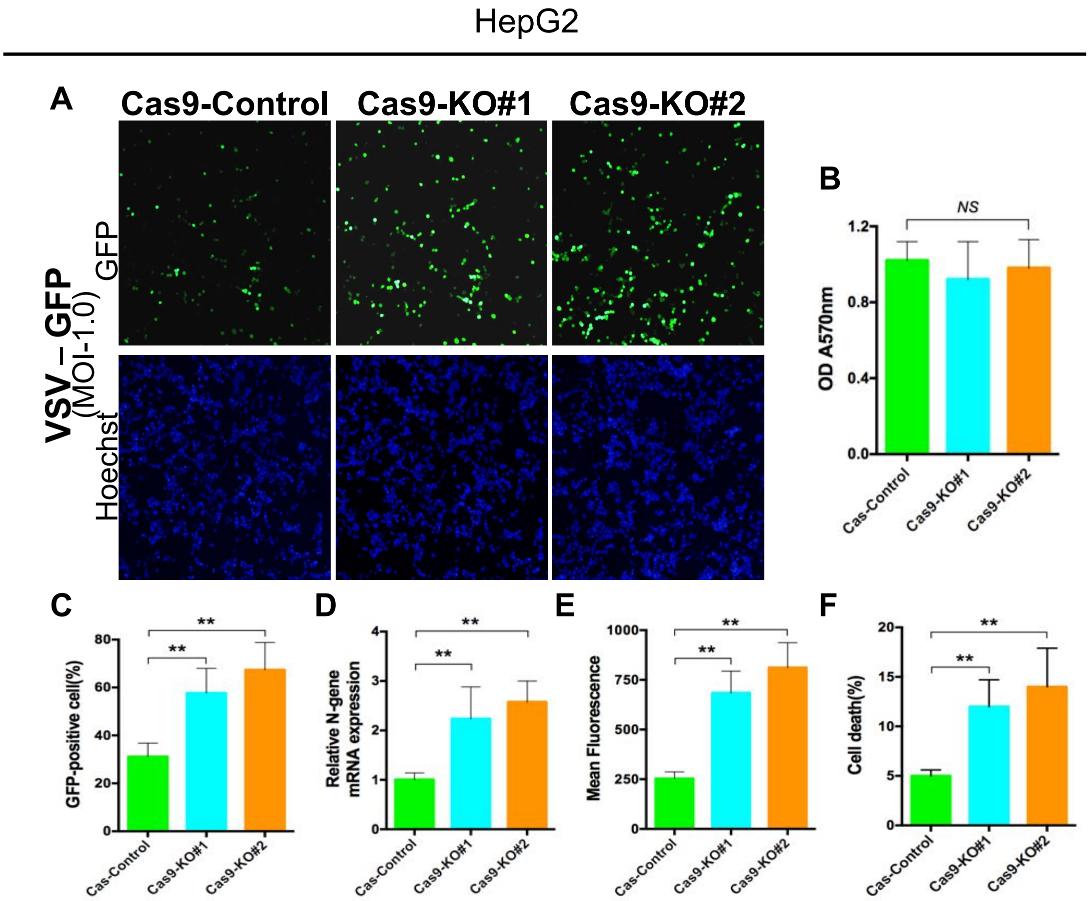

Fig.S5. Effects of c11orf83 deletion to rVSV-GFP replication in HepG2 cells. All analysis was performed at 12 hours post rVSV-GFP infection (MOI=0.1). **(A)** Representative imaging GFP fluorescent and Hoechst shows the replication of rVSV-GFP in cells. **(B)** Detection of cells viability using MTT. **(C,E)** Quantitative analysis of GFP positive cells and mean fluorescence intensity in cells by using flow cytometry. **(D)** Quantitative RT-PCR results showing the relative change of N gene transcription. **(F)** Quantitative analysis of VSV-induced cells death using Trypan Blue staining. **, $p<0.01$. NS, No Significance.

Supplementary Figure 6

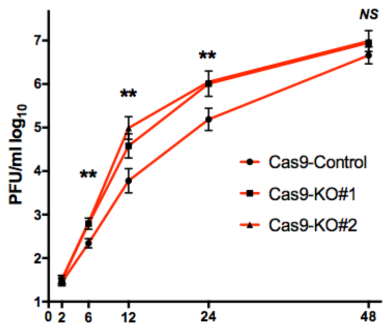

Fig.S6. Growth kinetic of VSV in HEK293 cells and C11orf83 KO cells. **, p<0.01.

Supplementary Figure 7

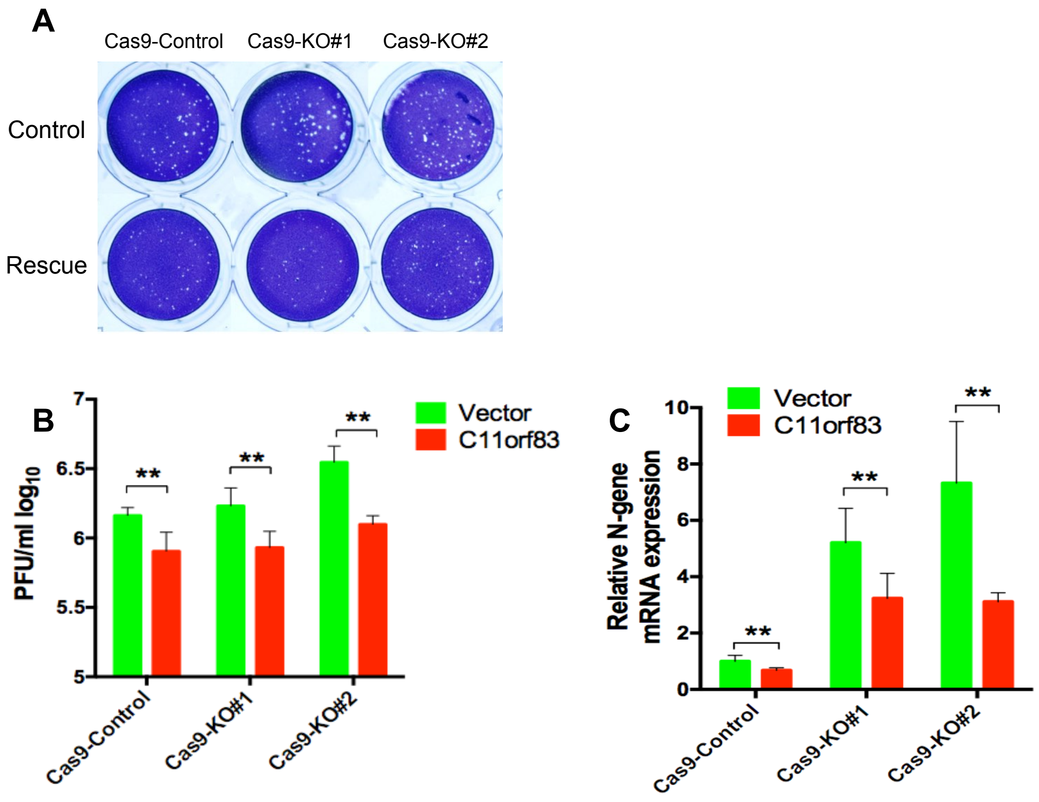

Fig.S7. (A,B) Rescue assay to analyze viral titers in C11orf83-deficient HEK293 cells by reconstituting C11orf83 expression. **(C)** Quantitative RT-PCR to detect N-gene transcription in C11orf83-deficient HEK293 cells that C11orf83 expression was rescued. **, p<0.01.

Supplementary Figure 8

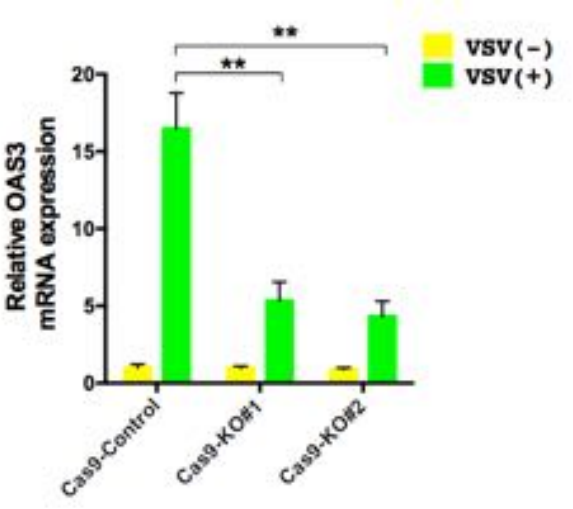

Fig.S8. Quantitative RT-PCR detection of OAS3 transcription in C11orf83-deficient HEK293 cells at basal levels and after VSV infection **, p<0.01.

Supplementary Figure 9

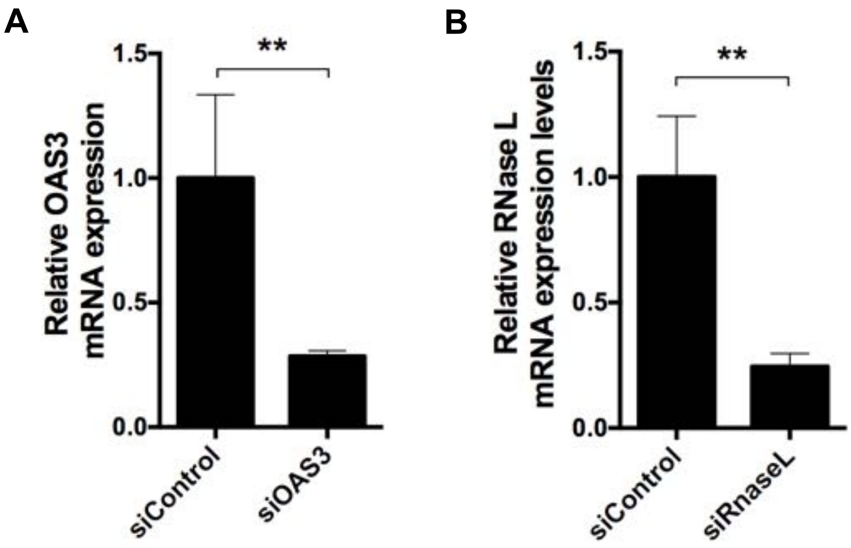

Fig.S9. (A,B) Silencing efficiency of siRNAs targeting OAS3 (A) and RNase L (B). **, p<0.01.

Supplementary Figure 10

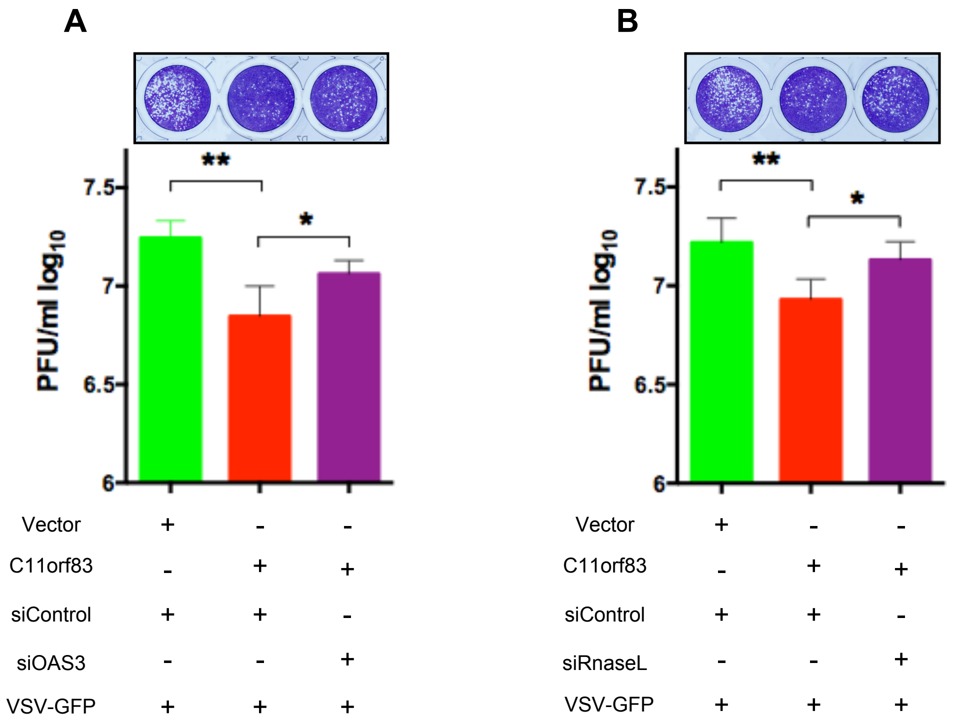

Fig.S10. Effects of OAS3 **(A)** and RNaseL **(B)** knockdown to rVSV-GFP replication in C11orf83-overexpressed HEK293 cells. **, p<0.01. *, p<0.05.

Supplementary Figure 11

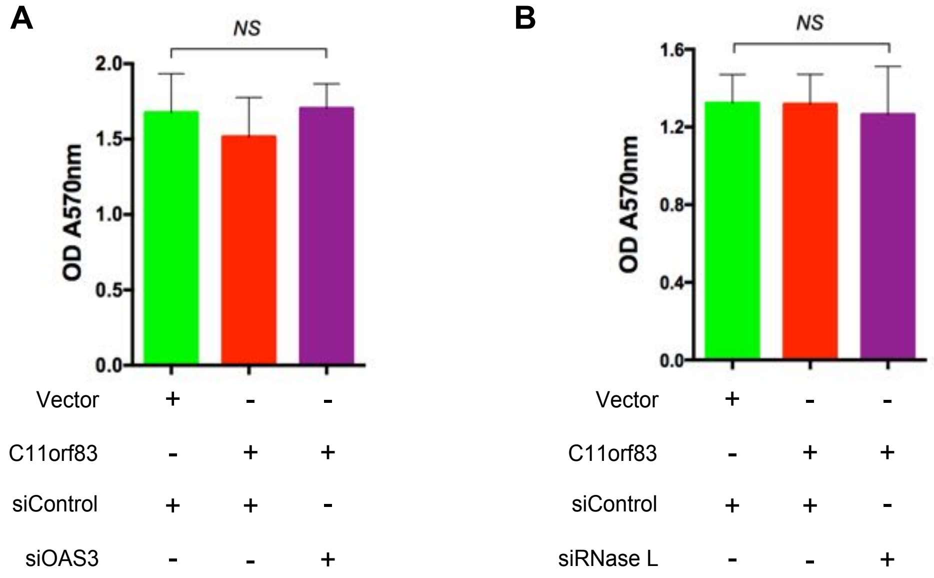

Fig.S11. MTT analysis of cells viability. HEK293 cells were transfected with pcDNA3.1 (vector) or pcDNA3.1-C11orf83, and 48 hours later cells were transfected with siControl, siOAS3 **(A)** or siRNase L **(B)**. NS, No Significance.

Supplementary Figure 12

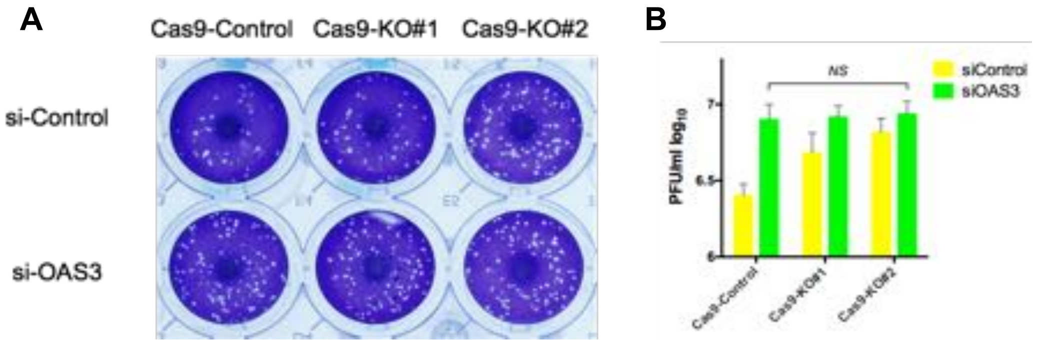

Fig.S12. (A) Plaque assay of viral titers in C11orf83-deficient HEK293 cells at 12 hours after VSV infection (MOI=0.1) with or without OAS3 knockdown. **(B)** Quantification of plaque assay to viral titers. **, p<0.01. NS, No Significance.

Supplementary Figure 13

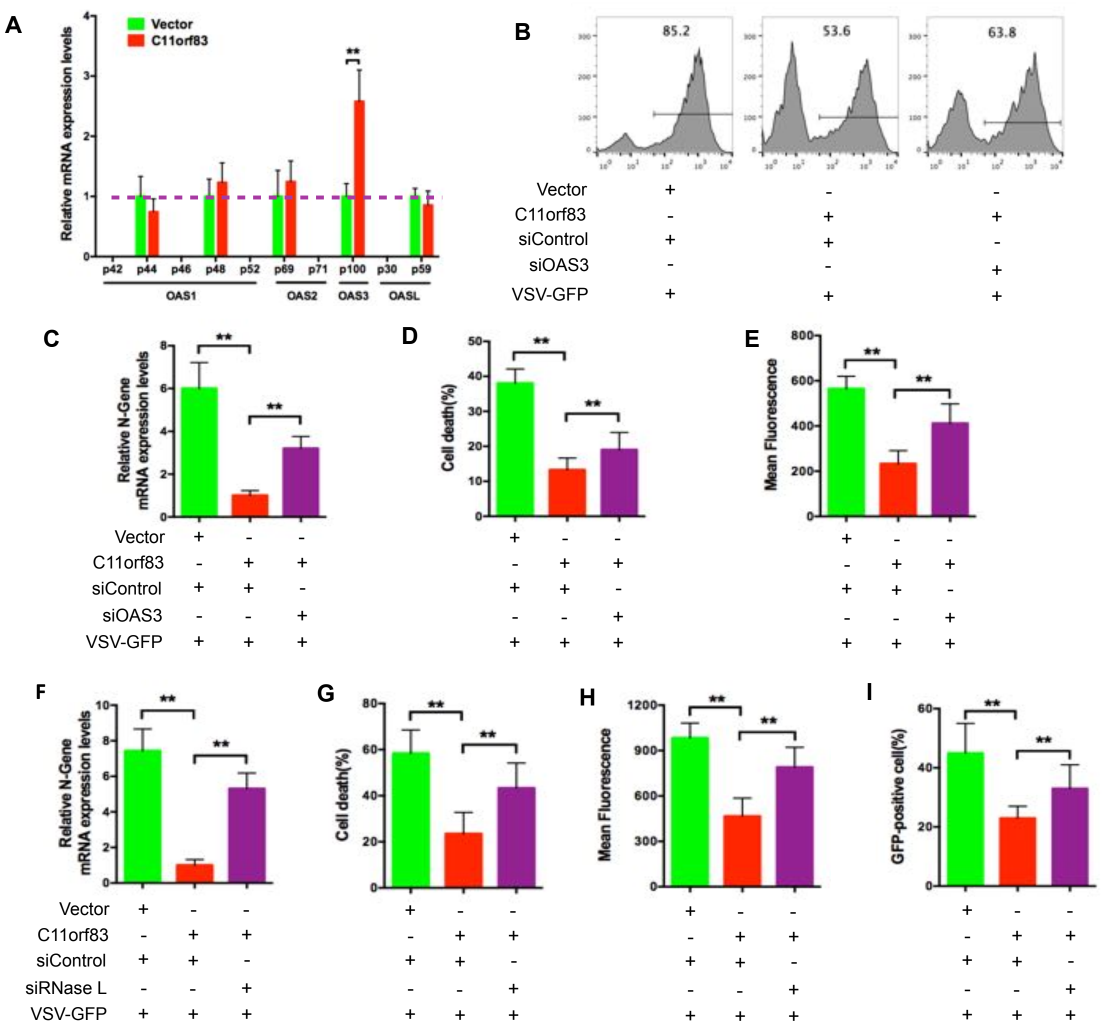

Fig.S13. (A) qPCR analysis of OASs in C11orf83 overexpressed HepG2 cells. **(B-E)** Effects of the loss of OAS3 to rVSV-GFP replication in C11orf83 overexpressed HepG2 cells. (B,E) Quantitative analysis of GFP positive cells and mean fluorescence intensity in cells by using flow cytometry. (C) Quantitative RT-PCR results showing the relative change of N gene transcription. (D) Quantitative analysis of VSV-induced cells death using Trypan Blue staining. **, $p<0.01$. (**F-I)** Effects of the loss of RNase L to rVSV-GFP replication in C11orf83 overexpressed HepG2 cells. **(F)** Quantitative RT-PCR results shows the relative change of N gene transcription. **(G)** Quantitative analysis of VSV-induced cells death using Trypan Blue staining. **(H,I)** Quantitative analysis of GFP positive cells and mean fluorescence intensity in cells by using flow cytometry. **, $p<0.01$.

Supplementary Figure 14

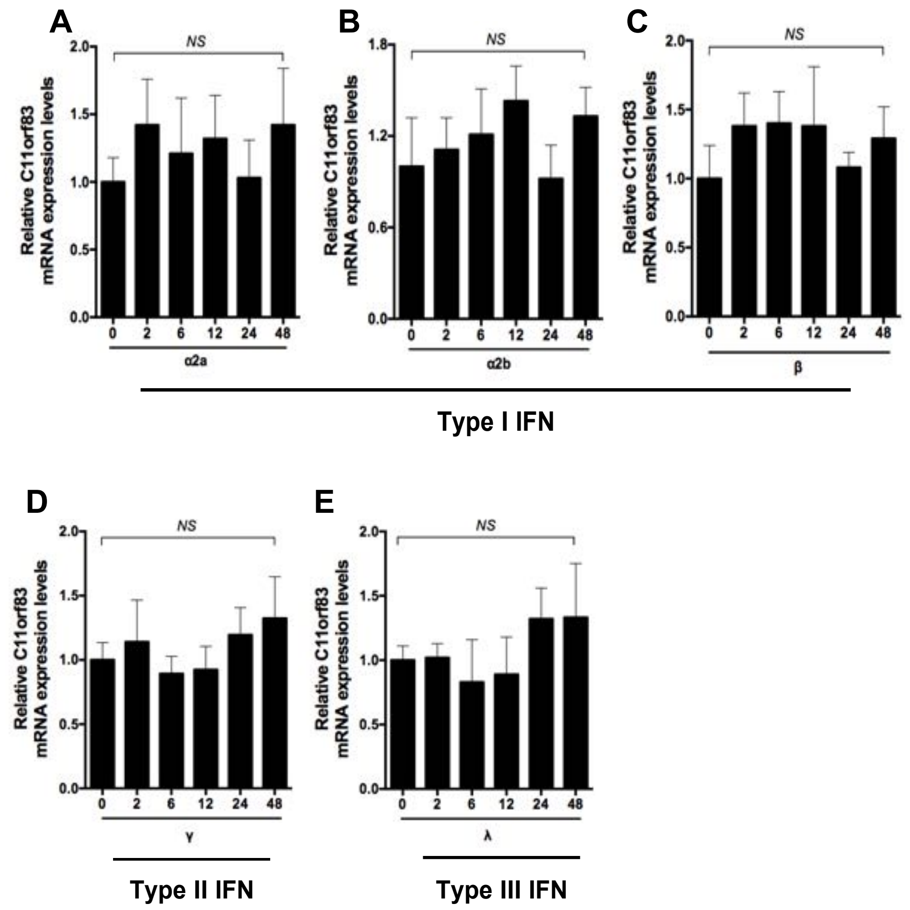

Fig.S14. Kinetics of C11orf83 expression upon type I (α2a,α2b and β), type II (γ)and type III(λ) IFN at 0,2,6,12,24 and 48 hours time post stimulation in HEK293 cells. NS, No Significance.

Supplementary Figure 15

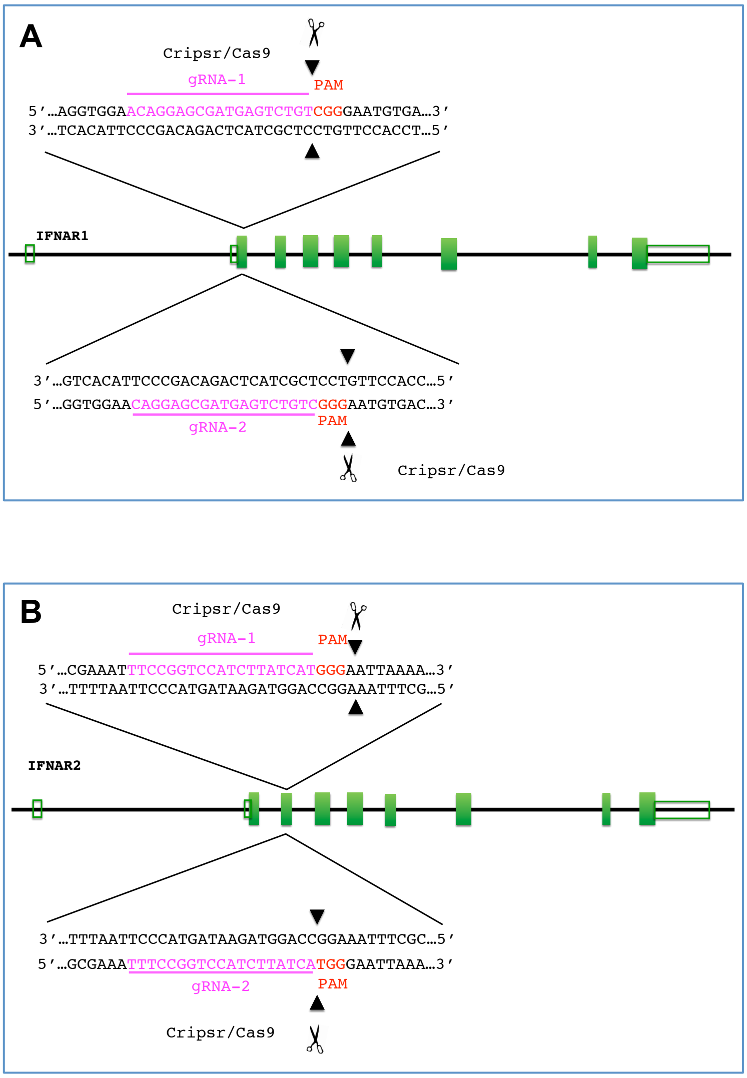

Fig.S15. Schematic diagram of CRISPR/Cas9 targetting IFNAR1 (A) and IFNAR2 (B) using two sgRNAs. Empty boxes represent non-coding exons, green boxes represent protein-coding exons, and straight line represents introns.

Supplementary Figure 16

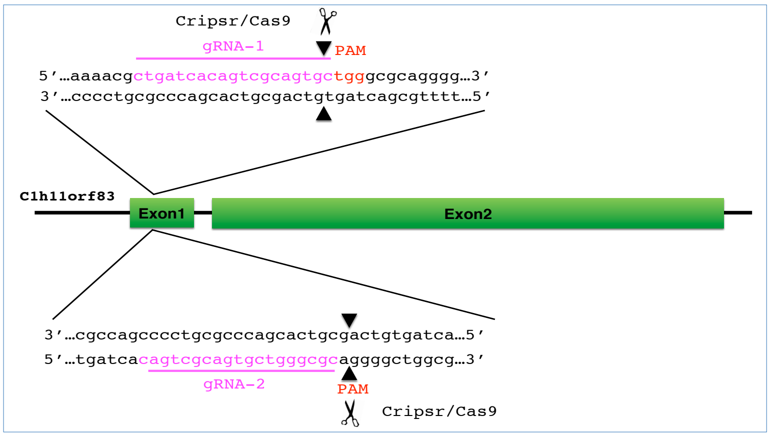

Fig.S16. Schematic diagram of CRISPR/Cas9 targeting monkey c11orf83 (C1H11orf83) using two sgRNAs in VERO cells. Empty boxes represent non-coding exons, green boxes represent protein-coding exons, and straight line represents introns.
